# MiR181-5p promotes pathogenic angiogenesis of hepatopulmonary syndrome by negatively regulating Wnt inhibitor Wif1

**DOI:** 10.22038/IJBMS.2023.70689.15362

**Published:** 2023

**Authors:** Dan Li, Guihua Li, Caiyi Li, Congwen Yang, Kaizhi Lu

**Affiliations:** 1 Department of Anesthesiology, Southwest Hospital, Third Military Medical University, Chongqing, 400038, China; 2 College of Traditional Chinese Medicine, Chongqing Medical University, Chongqing, 400016, China; # These authors contributed eqully to this work

**Keywords:** Angiogenesis, Hepatopulmonary – syndrome, MiR181-5p, Pulmonary microvascular - endothelial cells, Wnt inhibitory factor-1

## Abstract

**Objective(s):**

Hepatopulmonary syndrome is a serious respiratory injury caused by chronic liver disease. Excessive pulmonary capillary angiogenesis is the key pathological event. However, the mechanism of microRNA regulatory pulmonary capillary angiogenesis is still unclear.

**Materials and Methods:**

The hepatopulmonary syndrome rat model was constructed by Common bile duct ligation (CBDL) surgery. The expression tread of miR181-5p and Wif1 was detected by qRT-PCR and western blot in various tissues and disease processes. Wif1 was predicted as one of the potential target genes of miR181-5p by bioinformatic assay. miR181-5p mimics and inhibitors were used to increase/decrease miR181-5p levels in pulmonary microvascular cells. And Wif-1 specific recombinant lentiviruses were used to up-regulate and down-regulate Wif1 in pulmonary microvascular cells. Then, CCK8, Transwell, and tube formation assay were used for pulmonary microvascular cell proliferation, migration, and tube formation. And Dual-luciferase reporter assay was used to assess that miR181-5p may direct regulate Wif-1 in HPS rats.

**Results:**

The result showed miR181-5p specifically activates the Wnt signaling pathway by inhibiting Wif1 and then promotes pulmonary microvascular cell proliferation, migration, and tube formation, thereby accelerating the process of HPS. We finally verified Wif1 as a novel and direct target of miR181-5p in HPS.

**Conclusion:**

Taken together, we revealed an important miR-181-5p/Wif1/Wnt pathway in regulating pathological angiogenesis. It will prove beneficial as a therapeutic strategy for hepatopulmonary syndrome.

## Introduction

In China, there are more than 100 million hepatitis B virus carriers. Among them, approximately 20%–40% people developed hepatitis-cirrhosis. Seriously, 20%–30% of these hepatitis cirrhosis patients died because of Hepatopulmonary Syndrome (HPS), which significantly increases mortality (1, 2). The pathology of HPS includes alveolar capillary dilatation and pulmonary arteriovenous malformation (3, 4). Excessive alveolar capillary angiogenesis is the basis of capillary dilation and arteriovenous malformation. However, the mechanism is not clear. As we know miRNA can regulate gene expression by binding to the 3’ untranslated region (UTR) in a specific target gene, thereby repressing translation and/or degrading mRNA (5). miRNAs target a cluster of genes, allowing them to orchestrate biological processes widely (6, 7). Angiogenesis refers to the process of generating a neovascularization from the existing capillary network, including the formation, proliferation, and migration of endothelial spores, as well as the formation of vascular lumen and arteriovenous differentiation, and finally the formation of capillaries angiogenesis in cancer, vascular, rheumatoid and other diseases (8-10). Growing evidence suggests that miRNAs play an important role in the angiogenesis process. Certain miRNAs, such as miR144 miR-199, miR-206, and miR-9, have been shown to regulate the pathology of HPS (11-14). 

Wif1 is an important Wnt inhibitor that binds to Wnt ligands. It was the first identified to inhibit somitogenesis in Xenopus embryo development (15). Wnt signaling is vital for endothelial cell proliferation and angiogenesis. And the Wnt signaling pathway is also a direct mediator of endothelial cell growth and survival by VEGF, uPAR, FGF18, MMP3, and MMP7 (16-18). 

In this study, we found that the expression of miR-181-5p was significantly induced in the HPS process and miR181-5p promotes PMVECs proliferation, migration, and tube formation. Both *in vivo* and *in vitro* analyses demonstrated that transcription factor Wif1 is a key target gene of miR-181-5p. Our research indicates that miR-181-5p is involved in pathological angiogenesis by suppressing Wif1 in HPS. This study may provide a new strategy for HPS patient therapy.

## Materials and Methods

***HPS rat model***

The research was approved by the Animal Care Committee of Third Military Medical University (China). All experimental operations followed the guidelines from the National Institutes of Health. Male Sprague-Dawley rats (200-220 g) were purchased from the laboratory animal center of Third Military Medical University. Rats were maintained in disease-free conditions at 25 ℃, 55 ± 5% relative humidity, and a photoperiod of 12:12 hr (L:D). Before surgery, animals are free to eat standard pellet food and sterile water. Food was removed 8 hr before the operation. 

Common bile duct ligation (CBDL) operation was performed on the rats to construct the experimental HPS rat model as previously described (19). 15-40 mg/kg Pentobarbital Sodium was intraperitoneally injected for anesthetized rats. In the CBDL group, 30 rats underwent common bile duct ligation operation. In the sham group, 10 rats underwent common bile duct exposure but no ligation. For histopathological and qRT-PCR analysis, liver, lung, skin, stomach, intestine, spleen, and kidney was extracted from HPS and sham group rats after surgery 1w, 3w, and 5 weeks. At the same time, serum was collected from HPS and sham group samples (centrifugation at 2000 g, 4 °C overnight).

***Arterial blood gas analysis***

To avoid blood coagulation, a little heparin was absorbed in a 1 ml syringe to moisten the tube wall and drain it. The abdominal aorta of rats was exposed, as the abdominal aorta blood was collected as much as possible. 2 ml of blood was used to measuring arterial gas levels by PL2000 automatic blood gas analyzer according to standard operating procedures. PaCO_2_ and PaO_2 _of the rat were detected. Hypoxemia was defined as PaO_2_ <80 mmHg.

***Bioinformatics analysis***

MicroRNA181-5p potential target was predicted by the TargetScanHuman (https://www.targetscan.org/vert_71/).

*RNA isolation, cDNA synthesis, and qRT-PCR*

RNA isolation, cDNA synthesis, and qRT-PCR were according to previous studies (20). The total RNA of the tissues and cells was isolated by Trizol reagent (Invitrogen, USA). The DNA-free total RNA (2 μg per sample) was reverse-transcribed to cDNA using PrimeScriptTM RT reagent Kit with gDNA Eraser (Perfect Real Time, TaKaRa) in 20 μl. Quantitative real-time PCR was used to measure the transcription of candidate genes. Rat GAPDH was amplified as a control for each set of qRT-PCR reactions. The relative changes in gene expression were calculated by the 2^-ΔΔCt^ method. Primers designed by the Primer 5.0 software is listed in Supplementary Table S1.

*Protein isolation and Western blot*

The tissues and cells were lysed in RIPA lysis buffer (Beyotime, China), and then centrifuged at 12,000 g at 4 ℃ for 15 min. Then, supernatants were stored at -80 ℃. The same volume of protein from the lung of sham and HPS rats were loaded with SDS-PAGE gel (10 %) and transferred onto PVDF membranes (Millipore, USA). The membranes were blocked with 3 % skim milk for 2 hr. Membranes were incubated with primary antibodies against anti-Wif1 antibody (#ab155101, Abcam, USA) and used anti-β-actin antibody (#ab115777, Abcam, USA) as internal control overnight at 4 ℃. Then, the membranes were probed further with horseradish peroxidase-conjugated secondary anti-rabbit (#A0277, Beyotime, China) for 2 hr at room temperature. And visualized with PierceFase Western blot kit (Thermo Fisher Scientific, USA) and a cooled CCD camera system (Bio-Rad, USA).

*Histological analysis*

The sections were stained with hematoxylin and eosin (H&E) according to the standard protocol of the laboratory. And the morphology score was according to Cui *et al* (21).

*Immunofluorescence*

Fresh tissues were clipped and soaked in 4% paraformaldehyde for 6 hr 20% sucrose for 24 hr, and 40% sucrose for 24 hr. Make the tissue dry and soaking it in OCT for 30 min, cut it into 6 μm thick sections, and spread it on the slide. The frozen section was repaired in a quick antigen repair solution. Then, baked at 60 ℃ for 5 min; PBS for 5 min; blocked in 10% normal goat serum in a wet box on a slide for 60 min. then sections were incubated overnight at 4 °C with anti- (1:500, no. ab15580, Abcam), Then, sections were incubated in a 1:500 dilution of fluorescence-tagged secondary antibody for 2 hr. After washing three times with PBS, sections were incubated with DAPI for 10 min, and examined by fluorescence microscope.

*Vector construction*

The recombination vector was constructed for cell transfection assay. The nuclear fragment of rat Wif1 gene (NM_001399510) 3’ UTR region was amplified by PCR from the rat lung cDNA. And the fragment was cloned into pMD-19T simple vectors (TaKaRa Biotech, Japan). The combinational plasmid was digested by BamH I and Xba I after sequencing validation. Then, the 3’ UTR sequence of the Wif1 gene was transferred to the luciferase reporter plasmid pGL3 vector (Promega, USA). The pGL3 vector was modified by adding an SV40 promoter. The reporter vector was named pGL3-LUC-Wif1 UTR WT. 

The mutation vector with the miR181-5p binding site broken was constructed by the site-specific mutagenesis method. The whole vector was amplified by the special primer, which contains nucleic acids mutated in the target site. And pGL_3_-LUC-Wif1 UTR vector was used as a PCR template. Then, the vector was transformed into *Escherichia coli* competent cells. After sequencing validation, the plasmid was extracted for cell transfection assay. The mutative reporter vector was named pGL_3_-LUC-Wif1 UTR MUT. All of the cloning primers are listed in Table S1.

*Cell culture*

PMVECs were isolated from rat lungs as previously described(22). PMVECs were cultured in a primary endothelial cell medium provided by (iCell, SAIBAIKANG, PriMed-iCell-002), which contained some growth factors required for endothelial cell growth, such as VEGF, FGF, EGF, etc. And the medium was supplemented with 10 % fetal calf serum (FBS). The cells were cultured at 37 ℃ in a humidified atmosphere in 5 % CO_2_.

*Transfection and dual-luciferase reporter assay*

Vectors pGL3-LUC-Wif1 UTR (1 µg/ml) and pGL3-LUC-Wif1 UTR MUT (1 µg/ml) were transfected into PMVECs by Lipofectamine TM 3000 Transfection Reagent (Thermo Fisher Scientific, USA). The result of transfection was analyzed by the Dual-Luciferase Reporter assay System (Promega, USA) in the Glo Max-Multi Detection system Photometer (Promega, USA). miR181-5p mimic, miR181-5p inhibitor, control of mimic, and control of inhibitor were synthesized by Gene RIB Bio (Guangzhou, China). 

At 24 hr before transfection, cells were seeded in a 24-well plate as 1×10^5^ cells/well. miR181-5p mimic (50 nM) or miR181-5p inhibitor (100 nM) were transfected into the cells at 500 ng, together with 50 ng/well of pRL-TK (Promega, USA). After 24 hr of transfection, the luciferase activities were measured with a VARIOSKAN FLASH (Thermo Scientific, USA). The experiment was performed three times independently and the enzyme activity levels were presented as mean ± standard error.

*Wif1 overexpression and knockdown*

To analysis the function of Wif1 toward rat lung, a lentivirus with Wif1 overexpression and knockdown was constructed. The NC, Wif1 OE, and Wif1 KD viruses were treated PMVECs (each at 10 µl of 1×10^9 ^TU/ml). After 72 hr, detection of the Wif1 expression, CCK-8, Transwell, and Tube formation assays were carried on.

*CCK-8 assay*

Using Cell Counting Kit-8 (CCK-8; Beyotime, China) to evaluate cell proliferation as described(23). After serum treatment, cells were incubated with 10 μl CCK-8 solution in 96-well plates for 2 hr at 37 ℃. Living cells were detected by Variscan Flash multimodal reader (Thermo Fisher Scientific) in 450 nm taking absorbance. 

*Transwell assay*

The migration assay was as described (23, 24). Briefly, cells (1×10^5^) were seeded onto the upper chamber in 200 μl serum-free RPMI-1640 supplemented with 10 % FBS. After 24 hr of incubation, migrated cells on the lower surface of the filter were fixed and stained using crystal violet. Cells on the upper side were removed using a rubber scraper. Data represent counts of migrated cells. Experiments were performed in triplicate. 

*Tube formation assay*

Tube formation assay was according to the DeCicco-Skinner study (23, 25). Cool the 96-well plate and gun head in advance and prepare two precooled 1.5 ml centrifuge tubes for diluting Matrigel (BD, 356234). Matrigel was diluted in a 1:1 ratio of Matrigel to DMEM, then 50 µl Matrigel was added to each well to avoid bubbles. Place in an incubator at 37 ℃ for 45 min-1 hr. Cells were seeded in 96-well plates (3×10^4^/well). Tubules were photographed using phase microscopy after incubation for 0, 3, and 6 hr at 37 ℃ in a humidified atmosphere in 5 % CO_2_.

***Statistical analysis***

Statistical significance was calculated using Prism (GraphPad Software) and SPSS17. Data represented as mean± standard error. Statistical differences were assessed with two-tailed unpaired *t*-test, and *P-*values <0.05 were considered to reflect statistical significance.

## Results

***HPS rat model construction***

The previous research showed that miR-181 regulated endothelial cell dysfunction and tumor angiogenesis. In this study, we found the expression of miR181-5p was significantly enhanced with the HPS pathological progress. Therefore, we want to find out the relationship between miR181-5p and PMVECs proliferation and angiogenesis in HPS. 

**Figure 1:**
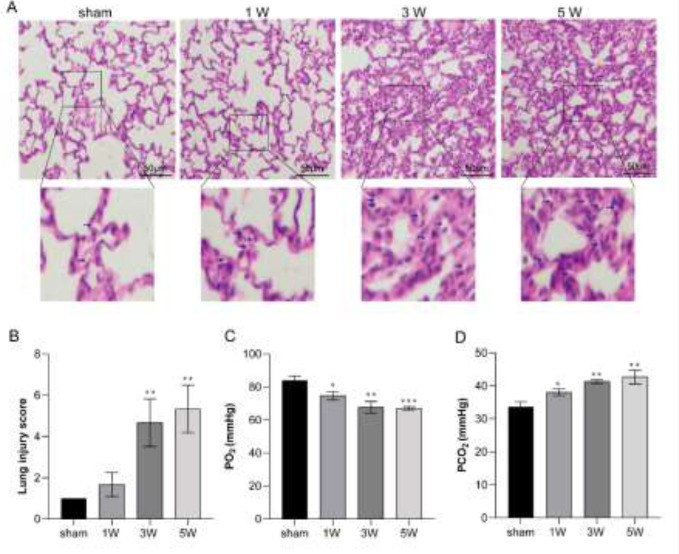
HPS rat model was constructed by common bile duct ligation (CBDL) surgery

Firstly, the experimental HPS rat model was constructed by the common bile duct ligation (CBDL). After surgery, H.E staining showed that the lung alveolar epithelial cells were flattened and the alveoli volume was bigger than that in the sham group (Figure 1A). And the lung injury score increased significantly when compared with that in sham group rats (Figure 1B). The PaO_2_ level of CBDL rats decreased gradually during the HPS pathological progress and PaCO_2_ level was opposite with that of the PaO_2 _(Figure 1C, D). 

***miR181-5p is upregulated in lung tissues of HPS rat***

**Figure 2:**
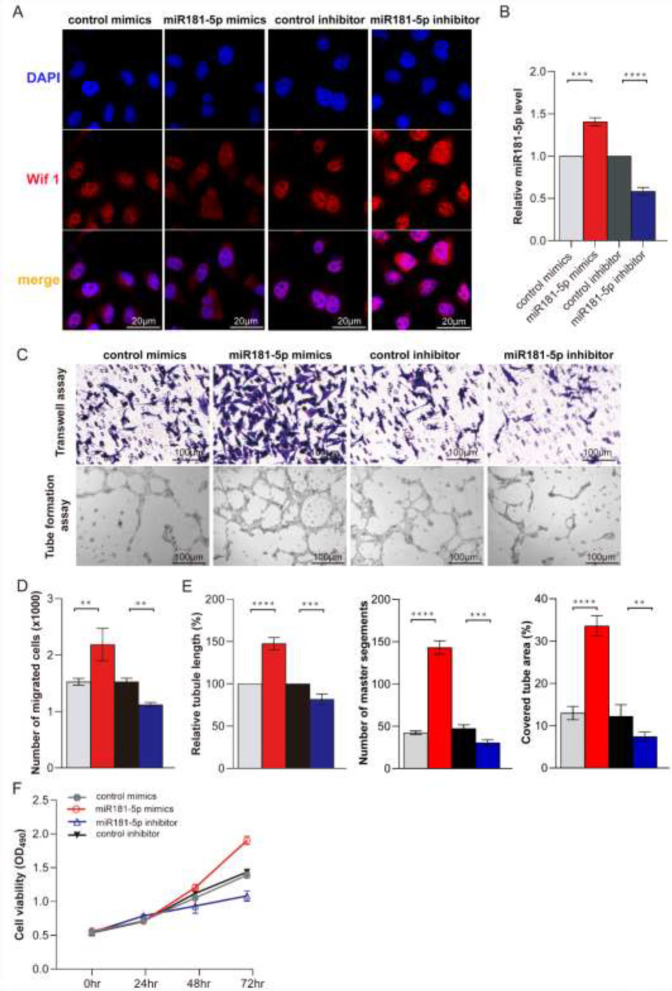
MiR181-5p is up-regulated in lung tissues of HPS rats

Profiling miRNA expression patterns in lung tissues of HPS rats and determining regulatory mechanisms of specific miRNAs enhance our understanding of molecular mechanisms of HPS. Based on our previous report of such profiling in HPS rats and normal subjects(11). We selected several miRNAs (8 upregulated and 8 downregulated) to determine their differential expressions in lung tissues from HPS rats and normal subjects. Compared with the sham group, miR124-5p, miR181-5p, miR10b-5p, miR199a-5p, miR708-5p, and miR883-3p were significantly upregulated, and miR181-5p was the most significantly upregulated with 2.7 folds. (Figure 2A-C). miR326-3p, miR1956-5p, miR3593-3p, miR26b-5p, miR126a-3p and miR144-3p were downregulated compared with sham group (Figure 2B). In this study, we identified the miR181-5p function in HPS. 

Then, the miR181-5p expression in the normal and CBDL rat lungs was detected. It showed that the miR181-5p level is highest in the lung than in other tissues (Figure 4A). And in the CBDL rat, miR181-5p expression is dramatically induced during the HPS rat pathological progression (Figure 4B). The results indicated that this molecular may regulate HPS progression.

***miR181-5p promotes PMVECs proliferation, migration, and tube formation***

**Figure 3:**
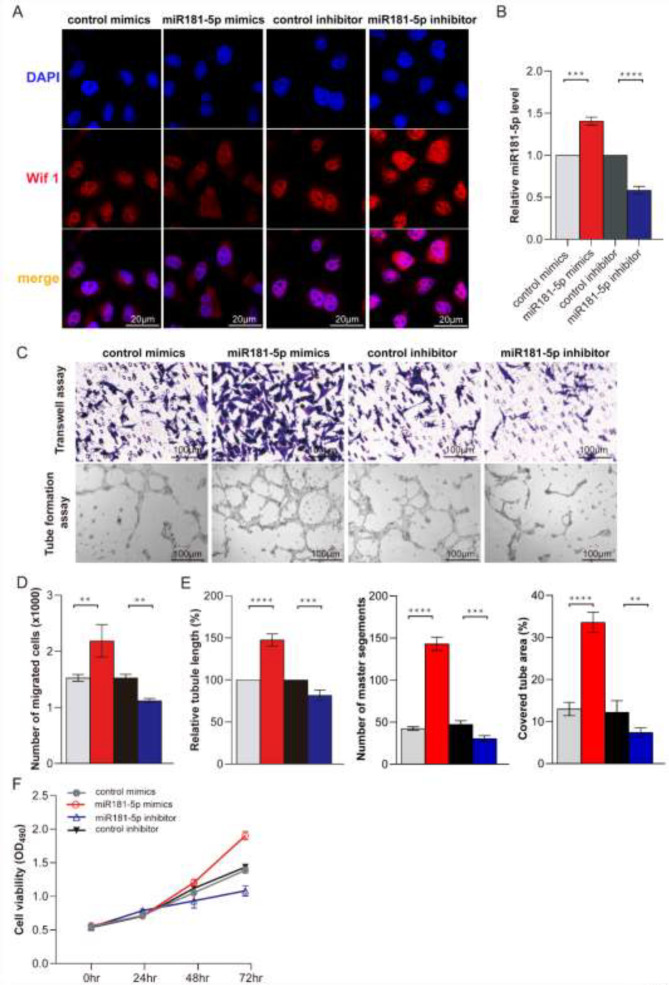
MiR181-5p promotes PMVECs proliferation, migration, and tube formation

To investigate the function of miR181-5p in pathological lung angiogenesis, we overexpressed or knocked down the miR181-5p expression in PMVECs using miR181-5p mimics and inhibitors, respectively. The result showed that miR181-5p mimic can increase miR181-5p 1.45 folds control that of the control mimics group. And miR181-5p inhibitor can reduce miR181-5p level to 65% that of the control inhibitor group (Figure 3A,B). Transwell and tube formation assay showed that miR181-5p mimic can promote PMVECs migration and tube formation (Figure 3C-E). However, miR181-5p inhibitor can restrain PMVECs migration and tube formation (Figure 3C-E). Furthermore, CCK8 assay showed that miR181-5p mimic promotes PMVECs proliferation and miR181-5p inhibitor restrained the PMVECs proliferation (Figure 3F). The data indicated that miR181-5p promotes PMVECs proliferation, migration, and tube formation. 

***Wif1 was a miRNA181-5p putative target gene***

The putative miR181-5p target genes were predicted by the microRNA online websites. There are total of 789 putative target genes that were predicted. Fifteen of them were transcriptional factors (Table 1). We found there is a miR181-5p binding site in the 3’UTR sequence of Wif1 (Wnt inhibitory factor-1) (Figure 6A). And the motif in the 3’UTR sequence of Wif1 gene was conserved across species (Figure 6D). It has reported that Wif1 can inhibit Wnt proteins, which are extracellular signaling molecules that play a role in embryonic development. Wnt signaling is essential for cell proliferation and angiogenesis (26, 27). The roles of Wnt signaling in some pathological conditions with abnormal neovascularization have been revealed recently (28). The role of Wnt signaling in vascularization has been established based on phenotypes of disruptions and mutations in the Wnt/Fz genes in animal models (29). Therefore, we hypothesized that Wif1 is one of the miR181-5p target genes to regulate pathological angiogenesis directly in HPS.

**Figure 4.**
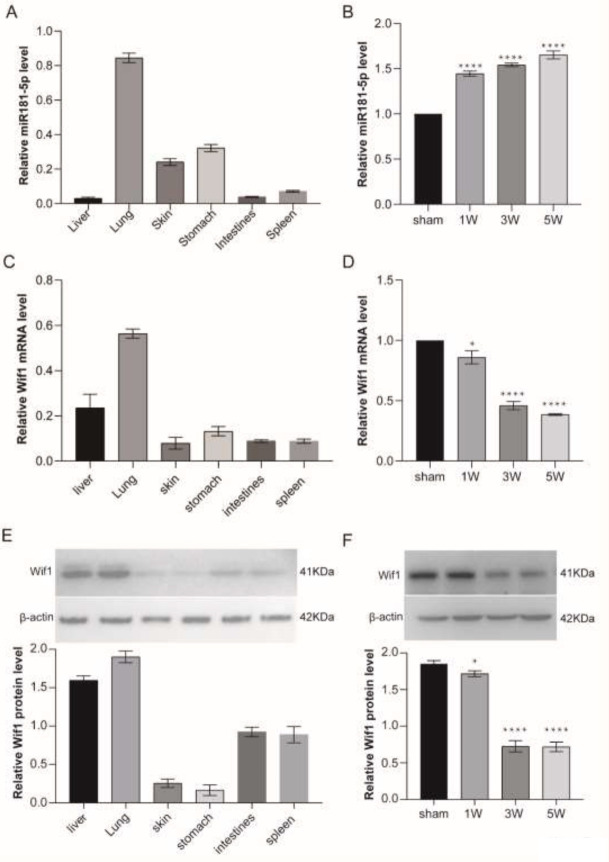
Wif1 expression trend was opposit that of miR181-5p during the HPS pathological progression

Then, we detected Wif1 expression in the normal rat and HPS rat models. QRT-PCR results showed that Wif1 was mainly expressed in the lung and liver of the normal rats (Figure 4C). And Wif1 expression decreased gradually during the pathological HPS progression (Figure 4D). Western blot validation of these results (Figure 4F). And the expression pattern of Wif1 is opposite to miR181-5p during the pathological HPS progression (Figure 4B). Furthermore, IF staining showed miR181-5p can inhibit or enhance Wif1 expression by adding miR181-5p specific mimics and inhibitor in PMVECs (Figure 3A). These data showed that Wif1 could be inhibited by miR181-5p specifically in PMVECs. 

***Wif1 inhibited PMVECs proliferation, migration, and tube formation***

**Figure 5:**
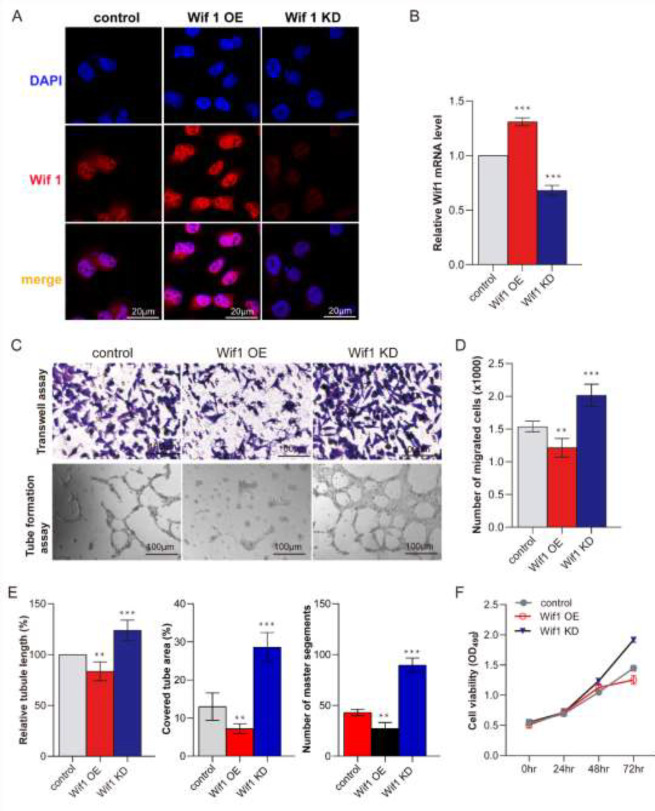
Overexpression of Wif1 inhibits PMVECs proliferation, migration, and tube formation

To investigate the function of Wif1, we use recombinant lentivirus to overexpression and knockdown Wif1 in PMVECs respectively. Firstly, IF staining showed that Wif1 protein synthesize was induced by Wif1 OE lentivirus, while Wif1 KD lentivirus reduced Wif1 protein expression (Figure 5A). QRT-PCR showed that Wif1 gene transcript induced by Wif1 OE lentivirus was 1.38-fold nearly that of the control group, and Wif1 gene transcript induced by Wif1 KD lentivirus was reduced to 72% of the control group (Figure 5B). Then, Transwell, tube formation, and CCK8 assay was used to assess the Wif1 gene function toward the PMVECs. It showed that Wif1 Overexpression inhibits PMVECs migration and tube formation. However, Wif1 knockdown induced PMVECs proliferation, migration, and tube formation (Figure 5C-E). Furthermore, the CCK8 assay showed that Wif1 overexpression inhibits PMVECs proliferation and Wif1 downregulation induced PMVECs proliferation (Figure 5F). These data showed that Wif1 is one of the miR181-5p putative target genes. And Wif1 overexpression inhibits PMVECs proliferation, migration, and tube formation.

***MiR181-5p inhibits Wif1 expression directly***

**Figure 6:**
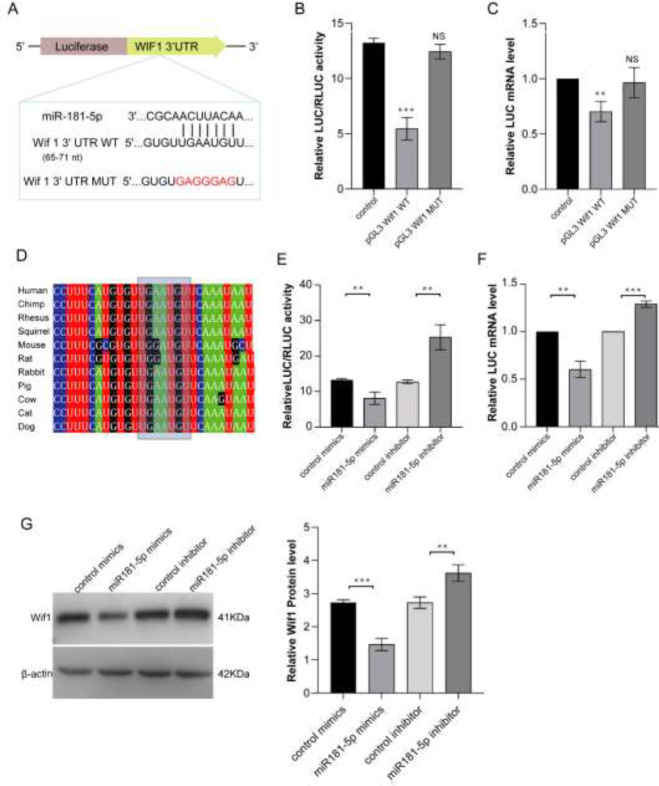
Identification of Wif1 as a direct target gene of miR181-5p in PMVECs

To identify that miR181-5p binds to the target sequence in the Wif1 3′ UTR, a dual luciferase reporter system was constructed. The pGL3 vector with Wif1 3′ UTR sequence of the LUC gene was transfected into the PMVECs. The cell was treated with an miR181-5p mimic and inhibitor to induce or reduce miR181-5p level in PMVECs. Then, the change in luciferase activity was assessed by dual-luciferase reporter assay. The result showed the relative Luc enzyme activity of the pGL3 vector with normal Wif1 3’UTR sequence was reduced to almost 75% than that of the control vector and the relative Luc enzyme activity of the pGL3 vector with mutant Wif1 3’UTR sequence has no significant difference from that of the control vector (Figure 6B). QRT-PCR detected the LUC gene expression and the result confirmed that of the Luc enzyme activity (Figure 6C). After miR181-5p mimic treatment, relative Luc enzyme activity was lower than that of control mimics treatment. miR181-5p inhibitor treatment, relative Luc enzyme activity was higher than that of control inhibitor treatment (Figure 6E). QRT-PCR detected the LUC gene expression and the result confirmed that of the Luc enzyme activity (Figure 6F). And Western blot showed Wif1 expression was inhibited after miR181-5p mimics treated PMVECs, and Wif1 expression was increased after miR181-5p inhibitor treated PMVECs (Figure 6G). These data showed that miR181-5p inhibits Wif1 expression by binding with the Wif1 3’UTR sequence directly. 

***MiRNA181-5p promotes angiogenesis by Wif1/Wnt/*** ***β*** ***-catenin pathway***

**Figure 7:**
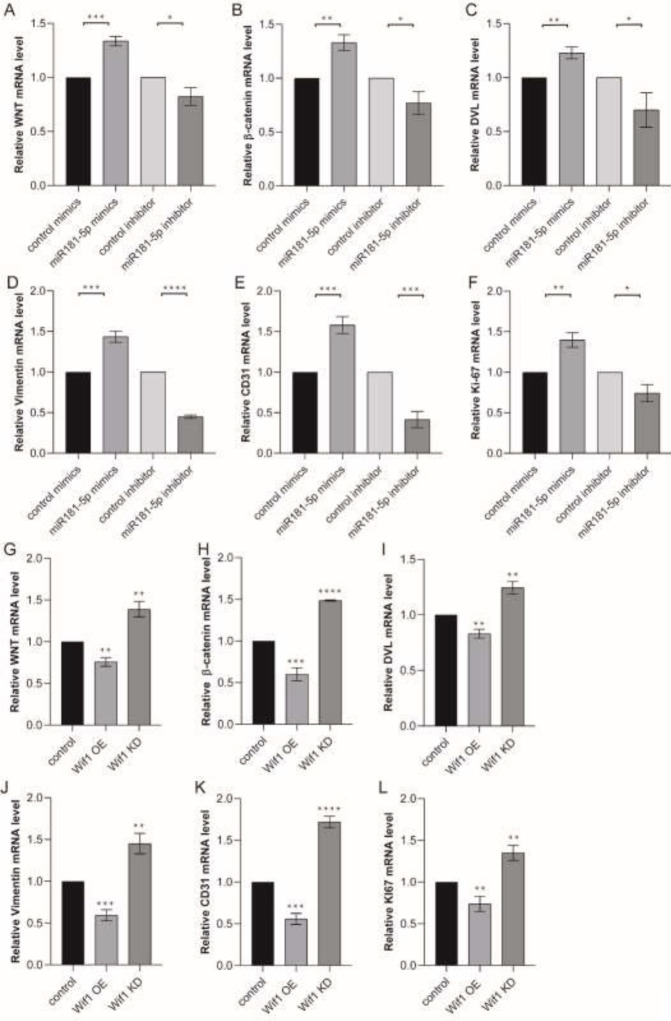
QRT-PCR detected Wif1/WNT pathway relative gene transcription in PMVECs

We detected the expression of genes in Wnt/ β catenin pathway and the genes which as angiogenesis markers during the miR181-5p mimic or inhibitor treatment in PMVECs. QRT-PCR showed Wnt, **β**-catenin, and DVL expression were induced by miR181-5p mimics treatment. And these gene transcriptions were reduced by miR181-5p inhibitor treatment (Figure 7A-C). Vimentin is an intermediate filament protein, CD31 is a blood vessel molecular marker and Ki67 is a molecular marker of cell proliferation. These genes expression was also induced by miR181-5p mimics treatment, reduced by miR181-5p inhibitor treatment (Figure 7D-F).

We also detected these gene expressions during the Wif1 OE or KD lentivirus treatment in PMVECs. QRT-PCR showed Wnt, β-catenin, and DVL expression was induced by Wif1 knockdown lentivirus treatment. And these gene transcriptions were reduced by Wif1 overexpression lentivirus treatment (Figure 7G-I). The results showed that miR181-5p promotes PMVECs proliferation, migration, and tube formation by inhibiting Wif1 expression. 

A) H&E staining of the lung in rats after CBDL surgery (n=30); B) Lung injury score of the HPS rat lung; C, D) PaO2 and PaCO_2_ level in HPS rat. (compared with sham group; *P<0.05, **P<0.005, ***P<0.001, and ****P<0.0001)

A) qRT-PCR detected up-regulated miRNA expression in HPS rat lung. B) qRT-PCR was detected to regulate miRNA expression in HPS rat lung. C) Fold change of differential miRNA expression in the lung of HPS rat. (n=3) (compared with sham group; *P<0.05, **P<0.005, ***P<0.001, and ****P<0.0001)

A) IF staining Wif1 expression in the PMVECs after treatment by miR181-5p mimics or inhibitor; B) qRT-PCR analysis of miR181-5p level during the treatment of miR181-5p mimic, mimic control, miR181-5p inhibitor, and inhibitor control; C) Transwell assay and tube formation assay analysis of PMVECs migration and the ability to form blood vessels during the treatment of miR181-5p mimic or miR181-5p inhibitor; D) Migrated cell statistic of transwell assay; E) Relative tubule length statistic of tube formation assay; F) CCK8 assay PMVECs proliferation during the treatment of miR181-5p mimic or inhibitor (n=3)

**Table 1:** putative target gene of miR181-5p

Target gene	Representative transcript	Gene name	Conserved sites total	Representative miRNA	Aggregate PCT
ZKSCAN1	ENST00000324306.6	zinc finger with KRAB and SCAN domains 1	1	hsa-miR-181-5p	0.31
CREBRF	ENST00000540014.1	CREB3 regulatory factor	2	hsa-miR-181-5p	0.93
KLF6	ENST00000542957.1	Kruppel-like factor 6	2	hsa-miR-181-5p	0.92
ZIK1	ENST00000307468.4	zinc finger protein interacting with K protein 1	2	hsa-miR-181-5p	< 0.1
ESM1	ENST00000381405.4	endothelial cell-specific molecule 1	1	hsa-miR-181-5p	0.71
BTBD3	ENST00000254977.3	BTB (POZ) domain containing 3	2	hsa-miR-181-5p	0.95
TNF	ENST00000449264.2	tumor necrosis factor	1	hsa-miR-181-5p	< 0.1
IL1A	ENST00000263339.3	interleukin 1, alpha	1	hsa-miR-181-5p	0.76
ZIC2	ENST00000376335.3	Zic family member 2	1	hsa-miR-181-5p	0.73
BCL2L11	ENST00000393256.3	BCL2-like 11 (apoptosis facilitator)	1	hsa-miR-181-5p	0.75
PARM1	ENST00000513238.1	prostate androgen-regulated mucin-like protein 1	1	hsa-miR-181-5p	0.75
IGF2BP3	ENST00000258729.3	insulin-like growth factor 2 mRNA binding protein 3	1	hsa-miR-181-5p	0.48
WIF1	ENST00000286574.4	WNT inhibitory factor 1	1	hsa-miR-181-5p	0.44
ARID2	ENST00000457135.1	AT rich interactive domain 2 (ARID, RFX-like)	1	hsa-miR-181-5p	0.7
EED	ENST00000327320.4	embryonic ectoderm development	1	hsa-miR-181-5p	0.76

A) qRT-PCR detected miR181-5p express in different tissues of normal rats; B) qRT-PCR detected miR181-5p express in the rat lung of different HPS pathological stages; C) qRT-PCR detected Wif1 express in different tissues of normal rat; D) qRT-PCR detected Wif1 express in the rat lung of different HPS pathological stage; E) Wif1 protein synthesize in different tissues of normal rat; F) Wif1 detected Wif1 protein synthesis in the rat lung of different HPS pathological stage (n=3)

A) IF staining Wif1 expression in the PMVECs after treatment by Wif1 OE or KD recombination virus; B) qRT-PCR analysis of Wif1 gene transcript; C) Transwell assay and tube formation assay analysis of PMVECs migration and tube formation during the treatment of Wif1 OE or KD recombination virus; D) Migrated cell statistics of transwell assay; E) Relative tubule length statistics of tube formation assay; F) CCK8 assay PMVECs proliferation during treatment of Wif1 OE or KD recombinant lentivirus (n=3)

A) Binding site of miR181-5p in the 3’UTR of Wif1. Red font indicates sequences that were mutated to abolish the interaction between miR181-5p and Wif1 3’UTR; B) Luciferase activity was analyzed 48 hr after PMVECs were con-transfected with pGL3-Wif1 WT or pGL3-Wif1 MUT; C) qRT-PCR analysis of relative LUC gene expression during cotransfection with pGL3-Wif1 WT or pGL3-Wif1 MUT; D) MiR181-5p binding site is highly conserved across species; E) Luciferase activity was analyzed after PMVECs were con-transfected with pGL3-Wif1 WT and miR181-5p mimic or inhibitor; F) qRT-PCR analysis of relative LUC gene expression during con-transfected with pGL3-Wif1 WT and miR181-5p mimic or inhibitor; G) Western blot analysis of Wif1 expression in the PMVECs after miR181-5p mimics or inhibitor treatment (n=3)

A–F) PMVECs were treated with miR181-5p mimic or inhibitor; G–L) PMVECs were treated with Wif1 OE or KD recombination virus (n=3)

## Discussion

Pathological pulmonary arterial smooth muscle cell (PASMCs) phenotypic transition and pathological pulmonary vein micro-vessels (PMVECs) lead to gas exchange and reduced substance exchange efficiency, which are the core pathological mechanisms of HPS[30-32]. microRNA plays an important role in HPS pathological progression. Our previous studies found that there were several miRNAs participated in HPS pathological process. Xu found that miR-9 regulated PASMCs myoid phenotypic modulation and proliferation (13). Chen found that miR-206 inhibited PASMCs proliferation by down-regulating ANXA2 gene expression (14). Zeng *et al* found that miR199a, miR144-3p, and miR145-5p regulated PMVECs proliferation and promoted the pathological angiogenesis of microvasculature (11, 12, 33). In this article, we found that miR181-5p promotes PMVECs proliferation, migration, and tube formation by Wnt/β-catenin. All of the research showed that more than one microRNA is involved in the regulation of HPS disease progression. The mechanism is complex and it is difficult to assess which molecule is more important.

MiR181 is involved in ontogeny, cell differentiation, proliferation, apoptosis, inflammation, and other physiological functions (34). It is also involved in the progression of many diseases (35-37). miR-181 is one of the essential angiogenic regulator in regulating endothelial cell gene expression and function (38). According to the prediction of bioinformatics, miR181-5p has 1367 potential target genes, and 70 % of these target genes are transcription factors, suggesting that miR181-5P further affects the synthesis of functional molecules by regulating the synthesis of transcription factors. It also shows the diversity of its role, but also shows its importance. In this study, miR181-5P inhibited Wif1 protein synthesis by binding to Wif1 3’ untranslated region and degrading it. Wif1 is a specific inhibitor of WNT, inhibiting the synthesis of downstream signaling molecules and signal transmission by inhibiting the activity of WNT. In the HPS model, the expression of miR181-5p increased with the progression of the disease, while Wif1 expression was inhibited. When Wif1 is inhibited, WNT/β-catenin signaling is activated, which promotes pathological microvascular hyperplasia. These data showed that miR181 promoted pathological pulmonary microvascular hyperplasia and accelerated the pathological process of HPS by inhibiting Wif1 expression.

The Wnt/β-catenin signaling pathway plays a very important role in regulating pathological angiogenesis of HPS. The Wnt gene was originally found in mouse breast cancer integrase-1 and drosophila wingless gene. The researchers named the two genes the Wnt gene because of their high similarity in function and structure (39, 40). The Wnt/β-catenin signaling pathway extensively regulates cell function, individual development, and disease progression. In tumors, abnormal activation of the Wnt/β-catenin signaling pathway is closely associated with increased morbidity, progression of malignant progression, occurrence of poor prognosis, and even increased cancer-related mortality (41). To date, many cancer therapies targeting the Wnt/β-catenin signaling pathway have been developed, which is believed to provide new opportunities for clinicians to develop more satisfactory and precise therapies for cancer patients with abnormal Wnt/β-catenin signaling pathways (42). Previous reports have not shown that miR181-5p promotes pathological pulmonary microvascular regeneration by regulating the proliferation and migration of PMVECs, nor has it been reported that miR 181-5p participates in the regulation of pathological pulmonary microvascular proliferation through the WNT signaling pathway. The results of this study showed that MIR181-5p was initiated by inhibiting WIF1 synthesis in HPS, and activated the Wnt signaling pathway to promote pathological angiogenesis and further promote the process of HPS disease. 

## Conclusion

Our results provide evidence in principle that microRNAs may be useful for the future development of novel therapeutic strategies for HPS. Nevertheless, it is necessary to use MIR181-5P knockout animal models for further studies, and in the next step, we will cooperate with hospitals to screen HPS patients, and use human samples to verify our findings and further clarify the specific mechanism of miRNA function.

## Authors’ Contributions

K L and CY designed this study. D L contributed to the material preparation and data collection. GL contributed to formal analysis. C Y wrote the original manuscript. D L and GL contributed to manuscript editing. K L and C Y was responsible for project administration and supervision. All authors have read and agreed to the published version of the manuscript.

## Funding

This project was supported by the National Natural Science Foundation of China [81800060), Natural Science Foundation of Chongqing, China [cstc2020jcyj-msxmX0361], The Science and Technology Project Affiliated to the Education Department of Chongqing Municipality [KJZD-K202215104] and Xinglin program of Chongqing TCM/TCM-integrated Key discipline [2021-ZDXK-yc05].

## Conflicts of Interest

The authors declare no conflict of interest.
